# Key Depression and Anxiety Symptoms in Hospice Patients: A Network Analysis Combined With Latent Profile Analysis

**DOI:** 10.1093/geroni/igaf122.1449

**Published:** 2025-12-31

**Authors:** Zhiqi Yi, Shuo Xu, Jing Zhao, Hongmei Wang

**Affiliations:** University of Kansas, Lawrence, Kansas, United States; Renmin University of China, Beijing, Beijing, China (People’s Republic); Beijing Sereniturn Palliative Care Development Center, Beijing, Beijing, China (People’s Republic); Beijing Sereniturn Palliative Care Development Center, Beijing, Beijing, China (People’s Republic)

## Abstract

Depression and anxiety are prevalent among hospice patients. A detailed understanding of the symptom comorbidity and key symptoms of depression and anxiety among Chinese hospice patients facilitate targeted interventions. This study investigates the depression and anxiety symptom network and compares networks in different symptom profiles in 388 Chinese hospice patients (Mean age=73.62). Patient Health Questionnaire-9 and the Seven-Item Generalized Anxiety Disorder Scale were used to measure depression and anxiety. Psychometric network analysis and latent profile analysis were conducted using R. Hopelessness and anhedonia in depression, and excessive worry and nervousness in anxiety symptoms were identified as the most central symptoms. Hopelessness, nervousness, and irritability were identified as the bridging symptoms. Latent profile analysis identified two profiles based on sixteen symptoms: “mild-symptom” and “severe-symptom” profiles. Significant global strength differences were found between the networks of mild-symptom profile and severe-symptom profile. In the mild-symptom network, hopelessness, excessive worry, reduced concentration, and anhedonia were the central symptoms, while excessive worry, insomnia, and hopelessness were the bridging symptoms. In the severe-symptom network, hopelessness, difficulty relaxing, reduced concentration, and nervousness were the central symptoms, while hopelessness, irritability, and nervousness were the bridging symptoms. These findings highlight that hopelessness should be the key intervention target to reduce the overall depression and anxiety symptoms in Chinese hospice patients, combining intervening in other key symptoms, including anhedonia, excessive worry, and nervousness. Intervening in hopelessness, nervousness, and irritability helps reduce the concurrence between depression and anxiety. Nuanced intervention strategies for hospice patients with different symptom severity should be adopted.

